# The influences of dormitory exercise on negative emotions among quarantined Chinese college students during the COVID-19 pandemic

**DOI:** 10.3389/fpsyt.2023.1243670

**Published:** 2023-08-24

**Authors:** Yu Zhang, Zehui Wen, Junying Li, Yongbin Shi, ShuQiao Meng

**Affiliations:** ^1^School of Physical Education and Sport, Henan University, Kaifeng, China; ^2^College of Physical Education, Yangzhou University, Yangzhou, China

**Keywords:** COVID-19 pandemic, dormitory exercise, negative emotions, college students, exercise intervention

## Abstract

**Objectives:**

This study explores the influences of different types of dormitory exercise on the negative emotions of quarantined Chinese college students during the COVID-19 pandemic.

**Methods:**

In a 3-week intervention, participants self-selected into a rope skipping group (RSG), an aerobic exercise group (AEG), and a resistance exercise group (REG), while participants in the control group (CG) engaged, by choice, in no physical exercise. Exercise frequency was three times a week for 45 min, with an intensity of 60–80% of maximum heart rate. Before and after the intervention, all participants completed the Self-rating Anxiety Scale (SAS) and the Self-rating Depression Scale (SDS). Student’s *t*-test was used for intra-group comparisons, while ANOVA was used for inter-group comparisons.

**Results:**

Within the three exercise groups, there were some differences in the decrease levels of anxiety and depression, statistically significant at (*p* < 0.05); by contrast, the levels of anxiety and depression slightly increased in the CG group, again with statistical significance (*p* < 0.05). Comparing each of the RSG, AEG, and REG with the CG, we found some statistically significant differences in decreasing levels of anxiety and depression (*p* < 0.05). A comparison of the RSG and REG with the AEG also revealed some significant differences in levels of anxiety and depression (*p* < 0.05). However, the RSG and REG did not significantly differ in levels of anxiety and depression (*p* < 0.05).

**Conclusion:**

Long-term and strict dormitory quarantine led to different levels of anxiety and depression among quarantined college students. The three different types of medium-intensity dormitory exercise evidently decreased anxiety and depression levels of quarantined college students, but aerobic exercise had the greatest influence, while rope skipping and resistance exercise had equivalent effects.

## 1. Introduction

In December 2019, the coronavirus disease (COVID-19) pandemic broke out worldwide (1–3). COVID-19 was characterized by strong infectivity, fast transmission, long duration, and a lack of specific drugs (4). Besides seriously threatening human life and health, it brought unprecedented challenges to public health systems and government capacity across the world (5, 6). To coordinate the Chinese government’s requirements of pandemic prevention and control on student campuses, Chinese universities strictly enforced management measures such as dormitory quarantine for students who had close or indirect contact with anyone confirmed to have the disease. Like home quarantine, dormitory quarantine restricts students’ actions with the goal of protecting public health (7). For students confined to their dormitory, activity space is very limited. While dormitory quarantine plays a vital role in preventing and controlling the spread of the virus, some studies have shown high prevalence rates of anxiety and depressive symptoms among Chinese college students during the COVID-19 pandemic (8, 9). This may be because adults in the emerging adulthood period (18–29 years old) are characterized by significant psychological changes, relatively vague self-awareness, and often high and unstable characteristics when facing unknown risks (10, 11). In addition, relevant studies indicate that prolonged closure and strict management measures led to different degrees of negative emotions among quarantined college students (12–15), among which anxiety and depression were prominent (16, 17). Anxiety, depression, and other negative emotions can lead to serious self-harmful behaviors, such as suicide, and trigger physiological reactions, such as crying (18, 19). According to statistics of the World Health Organization, the global burden of mental disorders has increased during the COVID-19 pandemic, with cases of major depression and anxiety rising by 28 and 26%, respectively (20). The *2022 Blue Book on National Depression* also points out that 50% of depression patients in China are school students, of which about 35.32% are aged 18–24 (21). Therefore, for anxiety, depression, and other negative emotions of quarantined college students during the COVID-19 pandemic, positive measures are needed to regulate and intervene.

Relevant studies have pointed out that proper, regular physical exercise can effectively promote mental health, regulate negative emotions (22–26), and relieve individuals’ temporary anxiety, depression, and other psychological problems caused by a specific time or situation (27–30). For example, Dong (31) found that physical exercise can alleviate anxiety, depression, and other unhealthy psychological states caused by public health emergencies such as Severe Acute Perspiratory Syndrome. Reigal (32) considered that moderate physical exercise could reduce anxiety and other psychological problems during COVID-19 lockdowns. Moreover, Maugeri and Li pointed out that engaging in a certain amount of physical exercise during the pandemic could reduce anxiety, depression, and other symptoms (33, 34). Many studies have also indicated that when the frequency of physical exercise reaches a certain level, different forms of physical exercise have different positive effects on individuals’ anxiety and depression (35–37). For instance, Sexton et al. (38) showed that both walking and jogging can significantly reduce levels of excessive anxiety and depression. Liu (39) also reported that mindfulness Tai Chi outperformed physical training in improving negative emotions such as anxiety and depression in students. In sum, many studies have shown, both before and during the pandemic, that physical exercise positively influences people’s mental health (40, 41).

Compared with the forms of physical exercise tested in previous studies, the dormitory exercise activities used in this study are carried out in a narrow and closed space. They represent a new form of physical exercise suitable for college students required to stay in their dormitory. Dormitory exercise means limited activity space. However, at present, research on dormitory exercise and negative emotions is relatively scarce. Therefore, considering the lockdown, limited space, few sports equipment and another actual situation, this study chose a new form of dormitory exercise including rope skipping, aerobic exercise and resistance exercise to intervene the being quarantined college students with anxiety and depression due to the measures of prevention and control during COVID-19. By exploring the influences of different types of dormitory exercise on the anxiety and depression of quarantined students, this study not only provides guidance for relevant departments and schools seeking to alleviate college students’ psychological health problems but also provides a scientific basis for formulating exercise prescriptions. We test three study hypotheses:

Hypothesis 1: Long terms of dormitory quarantine leads to different levels of anxiety and depression among college students.

Hypothesis 2: Medium-intensity dormitory exercise decreases levels of anxiety and depression in quarantined college students.

Hypothesis 3: Different types of dormitory exercise reduce levels of anxiety and depression to different extents in quarantined college students.

## 2. Materials and methods

### 2.1. Experimental design and participants

Owing to the Chinese government’s COVID-19 prevention and control requirements and the actual closure and control time which from October to November, the exercise intervention in this study just lasted 3 weeks. The experimental process included a pre-test and a post-test. The exercise intervention was run from October to November 2022 at the Minglun Campus of Henan University in Kaifeng, Henan Province, China. Participants were freshmen, sophomores, and juniors who were quarantined in their dormitories. We chose not to include senior and graduate students in the study because of internships, graduations, job searches, and other reasons. The Questionnaire Star platform was used to distribute questionnaires. After submitting the questionnaire, participants will receive an online red packet envelope of 5–10 yuan. Surveys were conducted three times during the experiment (Table 1).

**Table 1:** Participants with anxiety and depression in each study group.

Index/score	RSG		AEG		REG		CG	
	*n*	%	*n*	%	*n*	%	*n*	%
Anxiety 69≥x≥50	30	11.2	31	10.8	31	11.7	35	12.1
Depression 69≥x≥50	26	9.7	27	9.4	26	9.8	28	9.7

“*x*” represents the standard score on the SDS or SAS (as applicable); RSG, rope skipping group; AEG, aerobic exercise group; REG, resistance exercise group; and CG, control group.

The first survey measured quarantined college students’ intentions to exercise in their dormitories. A total of 1,207 questionnaires were distributed, and 1,110 valid questionnaires were returned. Since the exercise types in the intention questionnaire include rope skipping, aerobics exercise, resistance exercise and no physical exercise. Therefore, Among them, 268 students chose rope skipping, 287 chose aerobic exercise, 265 chose resistance exercise, and 290 students chose no physical exercise. The second survey was a pre-test of anxiety and depression levels among the 1,110 participants who validly responded to the first survey. We eliminated invalid questionnaires, such as those with random or repetitive answers, leaving 1,065 individuals who returned valid questionnaires, including 189 students with anxiety and 170 with depression. For ethical reasons, participants with serious mental illnesses could not be permitted to take part in the exercise intervention. Such individuals were identified by scores of 70 or more on the Self-rating Anxiety Scale (SAS) and/or the Self-rating Anxiety Scale (SDS). On this basis, 52 students with anxiety and 55 with depression were excluded after the first round of screening. The second round of screening eliminated participants who exercised less than once a week or quit during the exercise intervention. After both screening rounds, 127 individuals with anxiety and 107 with depression formed the study’s final sample. According to a test using G*Power software (version 3.1), our experimental study met the minimum sample size criterion. The third survey was a post-test of anxiety and depression levels after the exercise intervention.

Before the intervention, all participants were informed of the process and purpose of the experiment and other relevant information. Participants signed an informed consent form, and were assured that their survey data and relevant information would only be used for this study. In addition, all study procedures were approved by the Biomedical Research Ethics Subcommittee of Henan University.

### 2.2. Experimental groups

Based on the intention survey results, the pre-test results on the SAS and SDS, pandemic prevention and control policies of the Chinese government, and the actual situation on campus, we divided the 234 participants into four groups. The three experimental groups comprised a rope skipping group (RSG), an aerobic exercise group (AEG), and a resistance exercise group (REG). The other group was the control group (CG), whose members did not engage in any physical exercise during the intervention. The numbers of participants with anxiety in the RSG, AEG, REG, and CG were 30, 31, 31, and 35, respectively, while the numbers of participants with depression in the RSG, AEG, REG, and CG were 26, 27, 26, and 28, respectively.

### 2.3. Exercise intervention schemes

The strict closure and other quarantine measures provided favorable conditions for an experimental study. Exercise intervention schemes were implemented and movement teaching delivered via collective networks. The apps used included Zoom multi-person video software, Daily Rope Skipping software, Tik Tok, and Keep. During the exercise intervention, the time, frequency, and intensity of physical activities were strictly controlled (Table 2). Each activity lasted 45 min, including 5 min of warming up, 32 min of exercise, and 5 min of stretching. Exercise frequency was three times a week, and sessions were scheduled for 4:15–5:00 pm on Tuesday, Friday, and Sunday. Participants exercised at a medium intensity, keeping their heart rate within 60–80% of the predicted maximum. ActiGraph GT3X was used to monitor and control the heart rate. To test the feasibility of the exercise intervention schemes, we conducted a pre-experiment to monitor the heart-rate levels of students in each experimental group, randomly selecting 11, 12, and 12 students with anxiety in RSG, AEG, and REG and 9, 10, and 10 students with depression in RSG, AEG, and REG, respectively. The pre-experiment results showed that the schemes were completed and well accepted. Because in pre-experiment, over 80% of the participants successfully completed the exercise intervention.

**Table 2:** Details of three types of physical activity in the exercise intervention.

Program	Week	Frequency	Time (min)	Intensity	Experimental process	Software
Rope skipping	1	3	45	60% ~ 80% of predicted maximum heart rate	Exercise arrangements introduced and the attentions and actions of rope skipping explained. The attentions include standardizing movements and avoiding injuries as much as possible etc. during exercise. 5-min warm-up, 35-min exercise, 5-min stretching. Exercise had eight groups: the time of each group was 4 min and each interval between them was 25 s, respectively.	Zoom, Daily Rope Skipping
Aerobic exercise					Exercise arrangements introduced and the attentions and actions of deep squats, side rolls, open and closed jumps, burpee jumps, etc., explained. The attentions include standardizing movements and avoiding injuries as much as possible etc. during exercise. 5-min warm-up, 35-min exercise, 5-min stretching. Exercise had four groups: the time of each group and interval between groups were 8 and 1 min, respectively.	Zoom, Tik Tok
Resistance exercise					Exercise arrangements introduced and the attentions and actions of deep squats, sit-ups, push-ups, two-head lifts, flat supports, etc., explained. The attentions include standardizing movements and avoiding injuries as much as possible etc. during exercise. 5-min warm-up, 35-min activity, and 5-min stretching. Exercise had four groups: the time of each group was 8 min and each interval between them was 1 min, respectively.	Zoom, Keep
Rope skipping	2	3	45	60–80% of predicted maximum heart rate	Completed the exercise according to the requirements. The duration of the activity was 45 min, including a warm-up and stretching for 5 min each.	Zoom, Daily Rope Skipping
Aerobic exercise						Zoom, Tik Tok
Resistance exercise						Zoom, Keep
Rope skipping	3	3	45	60– 80% of predicted maximum heart rate	Completed the exercise according to the requirements. The duration of the activity was 45 min, including a warm-up and stretching for 5 min each.	Zoom, Daily Rope Skipping
Aerobic exercise						Zoom, Tik Tok
Resistance exercise						Zoom, Keep

### 2.4. Measures

#### 2.4.1. Dormitory exercise intention questionnaire

We compiled the intention questionnaire based on the International and Physical Activity Questionnaire Short Form (IPAQ-SF) and the 2020 National Fitness Survey of China. Its items covered intended exercise type, time, frequency, and intensity. The IPAQ-SF is commonly used to measure physical activity levels over the past week. It divides individual physical activity levels into three groups: low, medium, and high. The higher the score, the higher the level of physical activity. Given the lockdown, venue limitations (the area of every dormitory in Henan University is not exceeding 20 m^2^), lack of sports equipment, and other features of participating students’ actual situation, we focused on rope skipping, aerobic exercise, and resistance exercise in the intention questionnaire: all three activities were convenient and practicable for participants.

#### 2.4.2. Self-rating anxiety scale

The SAS was originally compiled by William Zung in 1971 (42) and is widely used for the self-assessment of college students’ anxiety. It comprises 15 forward-scored items and five reverse-scored items. Each item is answered on a four-point scale: 1 = “none or a little of the time”; 2 = “some of the time”; 3 = “a good part of the time”; and 4 = “most or all of the time.” The main statistical index of the SAS is the total score (standard score). The rough score (the rough score is the sum of the scores of all items in the scale) is multiplied by 1.25 and rounded to a whole number to produce the standard score. A standard score below 50 is considered normal, while a higher standard score denotes more serious anxiety symptoms. In this study, Cronbach’s coefficient of the SAS was 0.912.

#### 2.4.3. Self-rating depression scale

The SDS was also originally compiled by William Zung in 1965 (43). It comprises 10 forward-scored items and 10 reverse-scored items. Each item is answered on a four-point scale: 1 = “none or very little of the time”; 2 = “some of the time”; 3 = “a good part of the time”; and 4 = “most or all of the time.” The main statistical index of the SDS is the total score. The rough score is multiplied by 1.25 and rounded to a whole number to produce the standard score and the rough score is the total score obtained by adding all the items in the questionnaire. A standard score below 50 is considered normal, while a higher standard score denotes more serious depression symptoms. In this study, Cronbach’s coefficient of the SDS was 0.818.

#### 2.4.4. Quality control

In the early stage of questionnaire design, the researchers received special training to ensure the quality of the survey. When designing the two questionnaires, the survey’s purposes both were clearly marked in the guides of questionnaires’ using. The SAS and SDS both have high reliability and validity and are widely used internationally. To ensure that participants could honestly report their psychological status, responses were anonymized and no private information such as names and addresses were collected. Participants were asked to complete the questionnaires within 2 days after the questionnaires were issued for avoiding selection bias from extending the study period. In addition, to ensure the effectiveness of the exercise intervention, the researchers were asked to master the experimental process about what they need to do during intervention, when to start and finish the intervention and so on. When analyzing and processing statistical data after the exercise intervention, the researchers strictly followed the principles of authenticity and objectivity.

#### 2.4.5. Statistical analysis

SPSS 25.0 software was used for data analysis and processing. Student’s *t*-test was used for intra-group comparisons before and after the intervention, while ANOVA was used for an inter-group comparisons. If there were statistical differences in the indexes between different groups, the Bonferroni method was used for the post-test. In all analyses, p<0.05 was considered significant, while p<0.01 was considered very significant.

## 3. Results

### 3.1. Pre-test results for psychological status

Before the exercise intervention, levels of anxiety and depression were tested in the 1,110 students who chose different types of dormitory exercise. The results revealed that 189 students had anxiety and 170 had depression. Among them, 52 students with anxiety and 55 with depression had standard scores equal to or higher than 70 (Table 3).

**Table 3:** Pre-test results of psychological status among quarantined college students.

Index/score	*n*	Percentage
x<50	876	82.2
Anxiety 50≪x≪69	137	12.9
x≫70	52	4.9
x<50	895	84.0
Depression 50≪x≪69	115	10.8
x≫70	55	5.2

“*x*” represents the standard score of scale.

### 3.2. Within-group comparison of SAS and SDS scores before and after the exercise intervention

#### 3.2.1. SAS scores

Compared with the pre-test (Table 4), participants in the RSG, AEG, and REG all had lower anxiety scores in the post-test, and these differences were all statistically significant (p<0.05). By contrast, the anxiety score of participants in the CG was slightly higher in the post-test than in the pre-test, and this difference was statistically significant (p<0.05).

**Table 4:** Within-group comparison results for SAS scores before and after exercise intervention (*M* ± *SD*).

Group	*n*	Pre-test	Post-test	*t*	*p*
RSG	30	59.00±3.54	55.63±3.39	6.293	0.000
AEG	31	59.03±3.08	53.26±3.10	9.792	0.000
REG	31	59.00±2.91	56.65±2.97	5.014	0.000
CG	35	59.06±3.11	59.31±3.26	−2.491	0.018
F		0.003	20.473		
p		1.000	0.000		

RSG, rope skipping group; AEG, aerobic exercise group; REG, resistance exercise group; and CG, control group.

#### 3.2.2. SDS scores

As for anxiety, the depression scores of participants in the RSG, AEG, and REG were all lower in the post-test than in the pre-test, and these differences were all statistically significant (p<0.05). By contrast the depression score in the CG was slightly higher in the post-test than in the pre-test, and this difference was statistically significant (p<0.05).

### 3.3. Between-group comparison of the influences of dormitory exercises on anxiety and depression

#### 3.3.1. Impact on anxiety

Table 4 also shows that anxiety levels did not significantly differ between the RSG, AEG, REG, and CG before the exercise intervention (*F* = 0.003, p>0.05) but did significantly differ between the groups after the exercise intervention (*F* = 20.473, p<0.05). Using the Bonferroni method for back testing, we found that after the exercise intervention, the anxiety level decrease in the AEG was significantly greater than the decreases in the RSG, REG, and CG (*p* < 0.05). Moreover, the decreases in anxiety levels in the RSG and REG were also significantly greater than that in the CG (*p* < 0.05). However, there was no significant difference between the RSG and REG (p>0.05). Table 5 reports these results.

**Table 5:** Results of multiple *post hoc* comparisons of the differences in anxiety levels between pre-tests and post-tests.

Group	Group	Difference between mean values	Standard error	*p*
	AEG	2.375^*^	0.816	0.026
RSG	REG	−1.012	0.816	1.000
	CG	−3.681^**^	0.792	0.000
	RSG	−2.375^*^	0.816	0.026
AEG	REG	−3.387^**^	0.809	0.000
	CG	−6.056^**^	0.785	0.000
	RSG	1.012	0.816	1.000
REG	AEG	3.387^**^	0.809	0.000
	CG	−2.669^*^	0.785	0.005
	RSG	3.618^**^	0.792	0.000
CG	AEG	6.056^**^	0.785	0.000
	REG	2.669^*^	0.785	0.005

RSG, rope skipping group; AEG, aerobic exercise group; REG, resistance exercise group; CG, control group; ^*^p < 0.05, ^**^p < 0.01.

#### 3.3.2. Impact on depression

For depression levels, Table 6 shows that the RSG, AEG, REG, and CG did not significantly differ before the exercise intervention (*F* = 1.365, p>0.05) but did significantly differ post-intervention (*F* = 28.680, *p*<0.05). As Table 7 reports, the results from using the Bonferroni method for back testing revealed that after the exercise intervention, the depression level decrease in the AEG was significantly greater than the decreases in the RSG, REG, and CG (*p* < 0.05). Furthermore, the decreases in depression levels in the RSG and REG were also significantly greater than that in the CG (*p* < 0.05). However, we found no significant difference between the RSG and REG (p>0.05).

**Table 6:** Within-group comparison results for SDS scores before and after exercise intervention (*M* ± *SD*).

Group	*n*	Pre-test	Post-test	*t*	*p*
RSG	26	58.81±3.92	55.85±2.98	5.103	0.000
AEG	27	58.74±3.49	53.04±2.44	7.925	0.000
REG	26	58.85±3.56	55.92±2.42	4.605	0.000
CG	28	58.75±3.09	59.64±2.75	−3.45	0.002
F		0.005	28.680		
p		0.999	0.000		

RSG, rope skipping group; AEG, aerobic exercise group; REG, resistance exercise group; and CG, control group.

**Table 7:** Results of multiple *post hoc* comparisons of the differences in depression levels between pre-tests and post-tests.

Group	Group	Difference between mean values	Standard error	*p*
	AEG	2.809^*^	0.730	0.001
RSG	REG	−0.077	0.737	1.000
	CG	−3.797^**^	0.723	0.000
	RSG	−2.809^*^	0.730	0.001
AEG	REG	−2.886^*^	0.730	0.001
	CG	−6.606^**^	0.716	0.000
	RSG	0.077	0.737	1.000
REG	AEG	2.886^*^	0.730	0.001
	CG	−3.720^**^	0.723	0.000
	RSG	3.797^**^	0.723	0.000
CG	AEG	6.606^**^	0.716	0.000
	REG	3.720^**^	0.723	0.000

RSG, rope skipping group; AEG, aerobic exercise group; REG, resistance exercise group; CG, control group; ^*^p < 0.05, ^**^p < 0.01.

## 4. Discussion

### 4.1. Analysis of the psychological status of dormitory-quarantined college students

The results show that before the exercise intervention, about 17.7% of surveyed college students had anxiety, with mild to moderate anxiety in 12.9% and severe anxiety in 4.8%. Similarly, about 16% of surveyed college students had depression, with mild to moderate depression in 10.8% and severe depression in 5.2%. These findings indicated that long-term quarantining in dormitories led to different levels of anxiety and depression among college students, consistent with Hypothesis 1. Because compared with previous studies, there had a significant increase in levels of anxiety and depression in our study (44, 45). However, there were slight differences in the rates of anxiety and depression among college students (46–48), which might be related to their backgrounds, the research paradigm, and measurement tools. Moreover, our study was carried out at the end of 2022, 3 years after the outbreak of the pandemic. Consequently, participating students may have become more accustomed to actively cooperating with the relevant departments to take effective quarantine measures, based on a clear understanding of the pandemic. Nonetheless, being confined to a narrow and closed space for long periods may have led to some college students developing different levels of anxiety and depression. After the exercise intervention, levels of anxiety and depression decreased in all three experimental groups. Therefore, students should find better solutions to decrease levels of anxiety and depression and promote mental health development when facing the major public emergencies.

### 4.2. The influences of different types of dormitory exercises on anxiety and depression levels

After 3 weeks of exercise intervention, levels of anxiety and depression decreased in all three experimental groups while increasing slightly in the CG. These findings indicate that medium-intensity dormitory exercise decreased anxiety and depression levels of quarantined college students, consistent with Hypothesis 2 and with the results of previous studies (49, 50). From the perspective of physiology, dormitory exercise is a scientific form of physical exercise which has reasonable exercise time, intensity, frequency, and so on. Also it not only stimulates the secretion of epinephrine and catecholamine neurotransmitters in the brain but also make the hypothalamus and pituitary gland secrete endorphins, which can make people feel happy and reduce levels of anxiety and depression (51–53). From the perspective of psychology, dormitory exercise not only transfers and disperses negative emotions (stress, anxiety, depression, etc.) but also causes correct cognition and positive emotions, which improve an individual’s resistance to anxiety and depression (54, 55).

Our findings also indicate that increase levels of anxiety and depression might be caused by various factors. On the one hand, the outbreak of COVID-19 was severe and complex, and when individuals in the emerging adulthood period received and dealt with bad news about the pandemic, their levels of anxiety and depression tended to increase. Moreover, their negative emotions were compounded by worries about pandemic prevention and control, disappointment over lifting the closure management of school, and other related issues. On the other hand, when confined to a single narrow, closed environment for a long time, college students were more likely to suffer from anxiety, depression, and other negative emotions.

This study found that three different types of dormitory exercise—rope skipping, aerobic exercise, and resistance exercise—could all decrease the levels of anxiety and depression. Aerobic exercise had the greatest impact but there was no difference in impact between rope skipping and resistance exercise. These result are only partly consistent with Hypothesis 3. Compared to monotonous and repetitive rope skipping and resistance exercise, aerobic exercise combines elements such as gradual intensity, diverse movements, and lively music, which not only facilitate the development of physical health but also enhance the regulation of psychological emotions and stimulate conscious participation in exercise with a positive and happy mood. The lack of difference between the effects of rope skipping and resistance exercise might be explained by the relatively short duration of the exercise intervention. In summary, dormitory exercise as a new form of physical exercise can effectively decrease levels of anxiety and depression in college students.

### 4.3. Strengths, limitations, and research prospects

This study has the following strengths. Its topic is timely and innovative. As far as we know, this study is the first to investigate anxiety and depression levels of college students quarantined in dormitories during the prevention and control of COVID-19. Relatedly, it is the first to propose a new exercise intervention program suitable for exercise in a dormitory. In addition, the exercise intervention schemes were designed after considering the target group’s exercise intentions. And the exercise intervention schemes were implemented through an online sports app. The new form of physical exercise made great contributions to our study. Thirdly, we found the sociodemographic characteristics of students who were quarantined in dormitory during the COVID-19 epidemic and this finding was interesting. Finally, our study explored the influence of different dormitory physical activities on anxiety and depression, and the present findings of this study may be useful for people suffering from anxiety or/and depression to regulate and intervene.

However, this study also had some limitations. First, it lacks some related references about in narrow and closed dormitories to exercise and how this exercise affects negative emotions. Second, participants were only freshmen, sophomores, and juniors at one campus of a single university in China. Sample selection was uneven, and no senior and graduate students were included. Third, we did not include participants with severe anxiety or depression. Fourth, the requirements of COVID-19 prevention and control in China prevented us from implementing an offline exercise intervention. Finally, because the Chinese government issued reopening policies in November 2022, we lost the relatively perfect experimental conditions and could not carry out a longer exercise intervention. These factors may have influenced the study’s results.

To address these limitations and build on our findings, future studies should select different groups from different regions for exercise interventions, according to research needs, and extend the intervention time through artificial control. They should also focus on the influences of exercise intervention on students with severe anxiety and depression. Finally, future research should explore the differences between traditional forms of physical exercise and online physical exercise using the sports app.

## 5. Conclusion

This study yields three main conclusions:

1. During the prevention and control of COVID-19, prolonged quarantine in dormitories led to different levels of anxiety and depression among quarantined college students.
2. Medium-intensity dormitory exercise decreased the levels of anxiety and depression in quarantined college students.
3. Different types of dormitory exercise all effectively decreased anxiety and depression levels, but aerobic exercise performed best.

## Data availability statement

The original contributions presented in the study are included in the article/Supplementary material, further inquiries can be directed to the corresponding author.

## Author contributions

YZ and Z-HW: conceptualization, methodology, resources, and visualization. YZ, Z-HW, and Y-BS: data curation. Z-HW and J-YL: funding acquisition. YZ: investigation, supervision, and validation. YZ, Z-HW, and J-YL: project administration. YZ, Z-HW, Y-BS, and S-QM: software. YZ, Z-HW, J-YL, Y-BS, and S-QM: writing—original draft. All authors contributed to the article and approved the submitted version.

## Conflict of interest

The authors declare that the research was conducted in the absence of any commercial or financial relationships that could be construed as a potential conflict of interest.

## Publisher’s note

All claims expressed in this article are solely those of the authors and do not necessarily represent those of their affiliated organizations, or those of the publisher, the editors and the reviewers. Any product that may be evaluated in this article, or claim that may be made by its manufacturer, is not guaranteed or endorsed by the publisher.
