# Supplementary figures and images for: The influences of dormitory exercise on negative emotions among quarantined Chinese college students during the COVID-19 pandemic
Source: Front Psychiatry. 2023 Aug 24;14:1243670. doi: 10.3389/fpsyt.2023.1243670 (PMC10483225; doi:10.3389/fpsyt.2023.1243670)

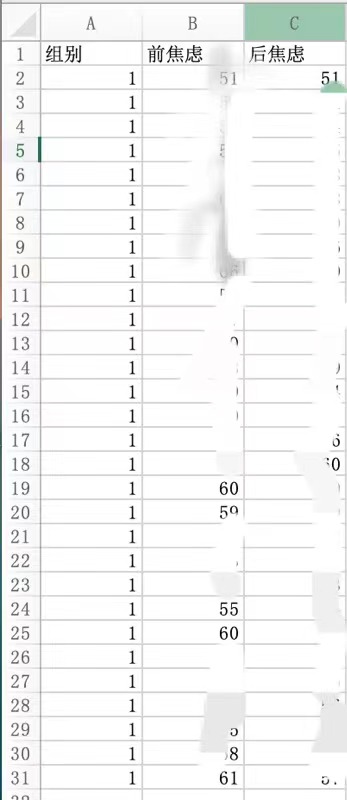

Supplement: Supplementary file 1 [file Image_1.JPEG]

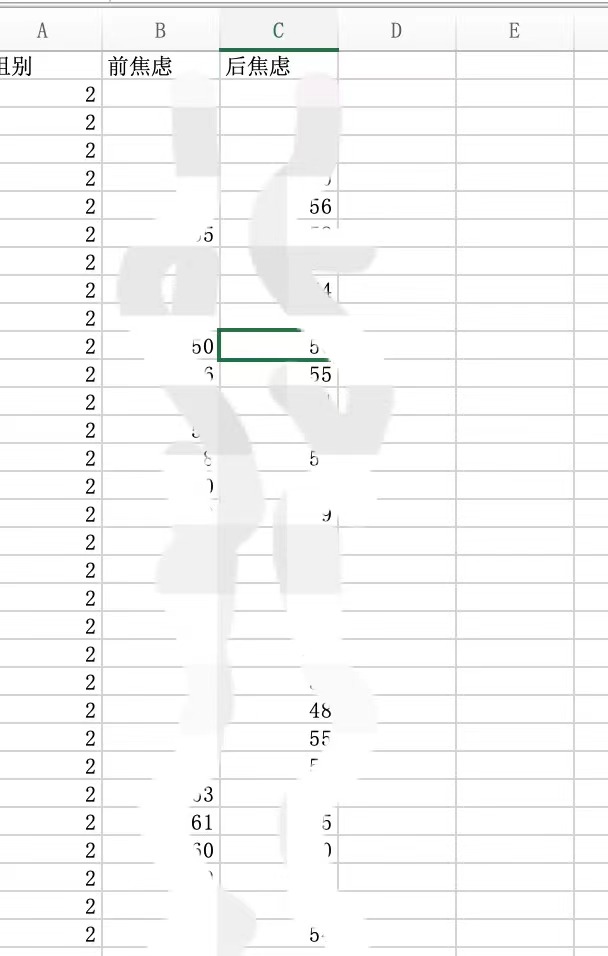

Supplement: Supplementary file 2 [file Image_2.JPEG]

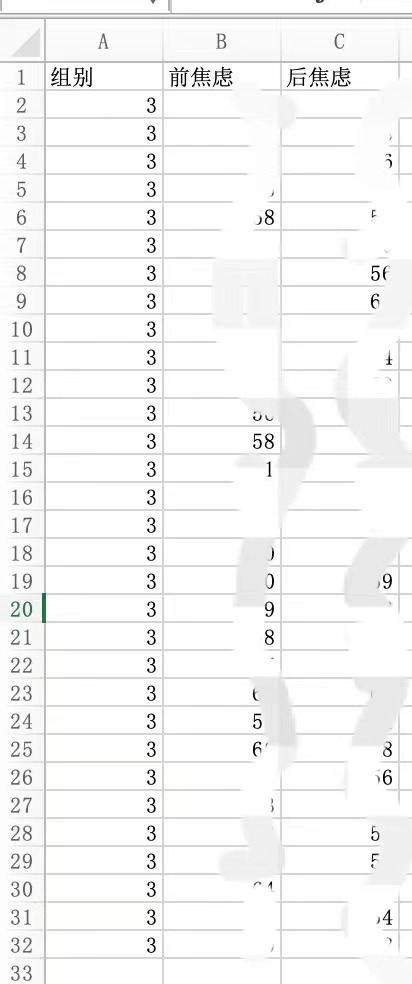

Supplement: Supplementary file 3 [file Image_3.JPEG]

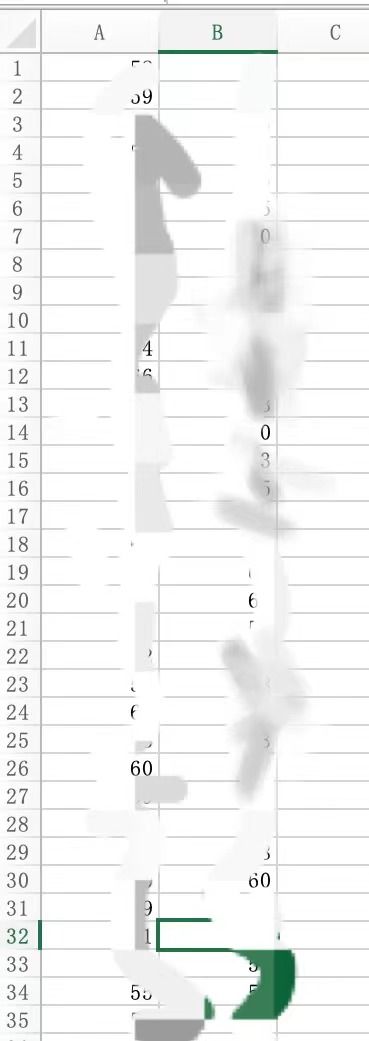

Supplement: Supplementary file 4 [file Image_4.JPEG]

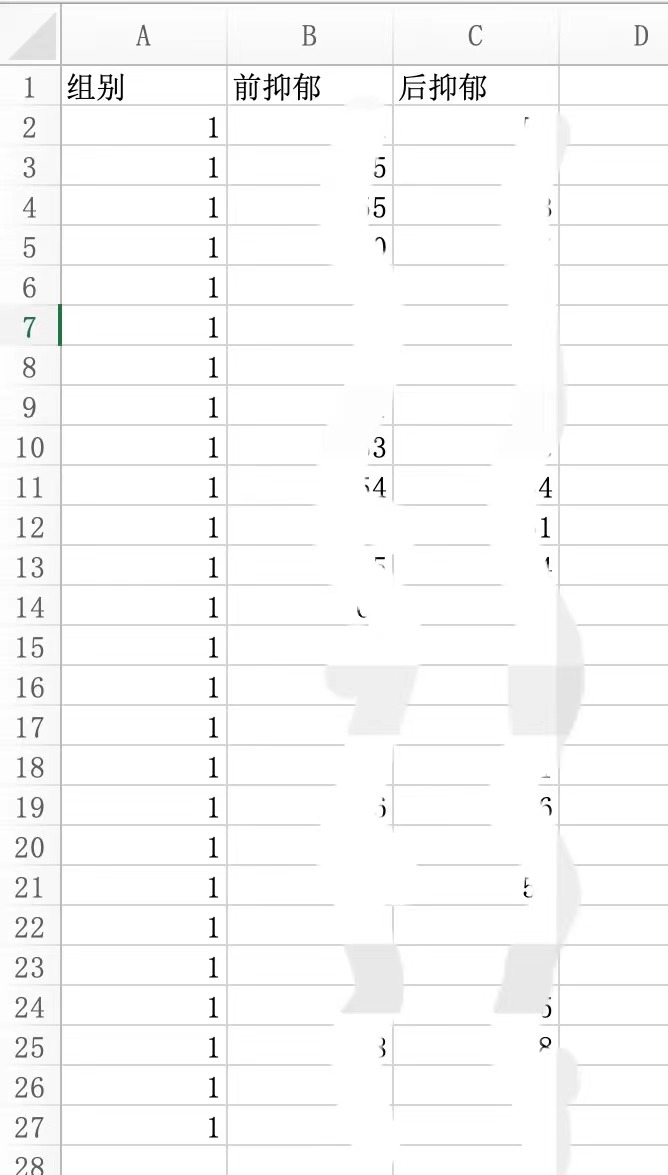

Supplement: Supplementary file 5 [file Image_5.JPEG]

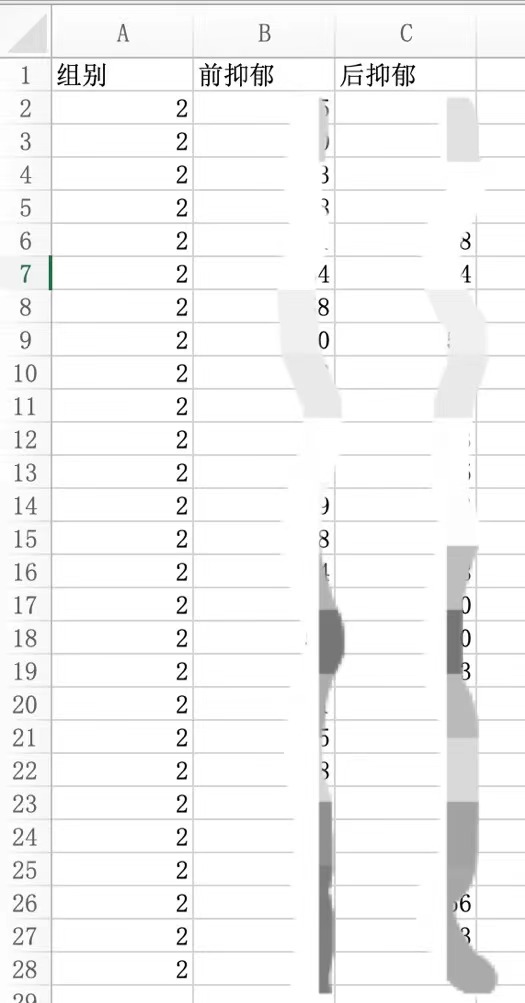

Supplement: Supplementary file 6 [file Image_6.JPEG]

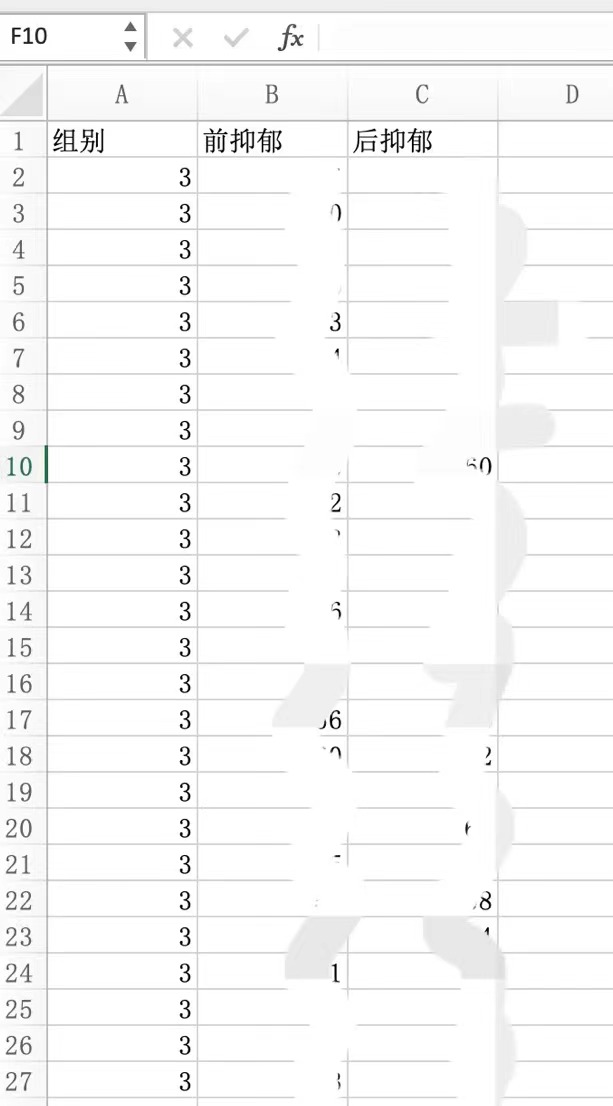

Supplement: Supplementary file 7 [file Image_7.JPEG]

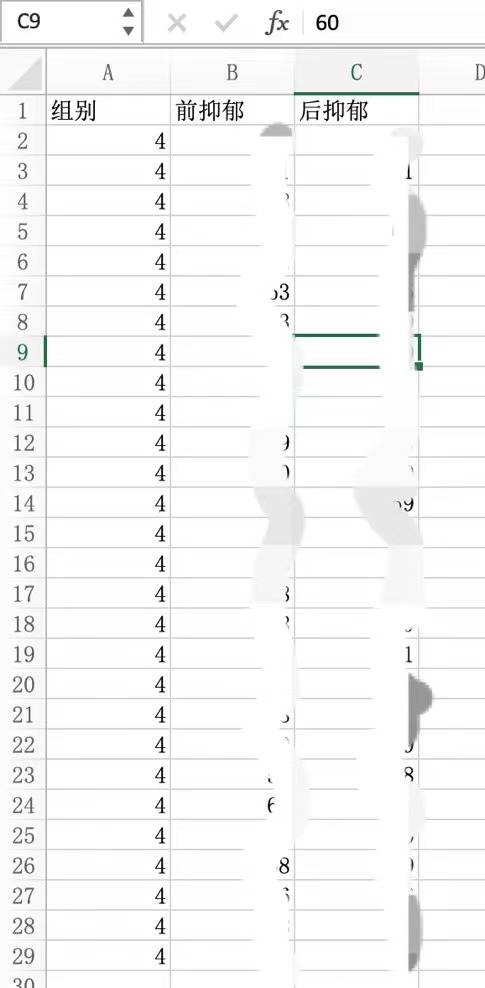

Supplement: Supplementary file 8 [file Image_8.JPEG]
